# Supplementary material for: Soluble amyloid-β precursor peptide does not regulate GABAB receptor activity
Source: eLife. 2023 Jan 23;12:e82082. doi: 10.7554/eLife.82082 (PMC9917443; doi:10.7554/eLife.82082)
Supplement: Figure 10—source data 2. [file elife-82082-fig10-data2.docx]

| Perfusion condition | Perfusion condition | p-value |
| --- | --- | --- |
| ACSF I | sc-APP17 | 0.6055 |
| ACSF I | ACSF II | 0.1222 |
| ACSF I | APP17 | 0.1995 |
| ACSF I | ACSF III | 0.3625 |
| ACSF I | baclofen | 0.0000 |
| sc-APP17 | ACSF II | 0.9156 |
| sc-APP17 | APP17 | 0.9756 |
| sc-APP17 | ACSF III | 0.9980 |
| sc-APP17 | baclofen | 0.0000 |
| ACSF II | APP17 | 0.9998 |
| ACSF II | ACSF III | 0.9936 |
| ACSF II | baclofen | 0.0000 |
| APP17 | ACSF III | 0.9997 |
| APP17 | baclofen | 0.0000 |
| ACSF III | baclofen | 0.0000 |

Statistical analysis between perfusion conditions in two-photon Ca^2+^ imaging experiments shown in Figure 10.
